# Training-of-trainers: A strategy to build country capacity for SLMTA expansion and sustainability

**DOI:** 10.4102/ajlm.v3i2.196

**Published:** 2014-09-16

**Authors:** Talkmore Maruta, Katy Yao, Nqobile Ndlovu, Sikhulile Moyo

**Affiliations:** 1 Clinton Health Access Initiative, Maseru, Lesotho; 2 International Laboratory Branch, Division of Global HIV/AIDS, Center for Global Health, US Centers for Diseases Control and Prevention, United States; 3 African Field Epidemiology Network, Kampala, Uganda; 4 Botswana–Harvard AIDS Institute Partnerships, Botswana–Harvard HIV Reference Laboratory, Botswana

## Abstract

**Background:**

The Strengthening Laboratory Management Toward Accreditation (SLMTA) programme uses a training-of-trainers (TOT) model to build capacity for programme scale-up. The TOT strategy is designed to maximise utilisation of its graduates whilst minimising inconsistencies and ensuring high programme quality during global expansion.

**Objectives:**

To describe the SLMTA TOT programme approach.

**Methods:**

The two-week training, led by carefully selected and trained master trainers, enables effective and authentic implementation of the curriculum by its graduates. The teachback methodology used allows participants to practise teaching the curriculum whilst learning its content. A trainer’s toolkit provides all the materials necessary for teaching and must be followed faithfully during training. Two surveys were conducted to assess the effectiveness of the TOT strategy: one sent to 316 TOT graduates in 25 countries and the other sent to the programme leaders in 10 countries.

**Results:**

By the end of 2013, 433 SLMTA trainers had been trained who, in turn, taught more than 1900 people to implement SLMTA in 617 laboratories in 47 countries. Ninety-seven percent of the 433 TOT graduates and 87% of the 38 master trainers are based in developing countries. Ninety-two per cent of the graduates have been utilised at least once in programme implementation and, as of August 2013, 87% of them were still actively involved in programme activities. Ninety-seven per cent of the graduates stated that the TOT workshop prepared them well for training or other programme tasks.

**Conclusion:**

The SLMTA TOT strategy is effective in building local capacity for global programme expansion whilst maintaining programme quality.

## Introduction

The current drive toward laboratory quality improvement and accreditation in resource-limited settings will be hard to sustain without building local capacity. Launched in 2009, the Strengthening Laboratory Management Toward Accreditation (SLMTA) programme has revolutionised the ability of government medical laboratories in low-resource settings to implement quality management systems and pursue international accreditation.^1,2^ SLMTA is an innovative management training programme that employs a series of workshops and improvement projects to realise immediate and measurable improvement.^1^ Within five years of its launch, SLMTA has been implemented in 617 laboratories from 47 countries in Africa, Southeast Asia, the Caribbean and Latin America and has been used to train more than 1900 people.^2^ Its rapid expansion is attributed in part to its effective local capacity-building effort using a training-of-trainers (TOT) strategy.

TOT has been applied across many disciplines including education, healthcare, health promotion and disease prevention to provide would-be trainers with the necessary knowledge and skills for training others.^3,4^ Its popularity is because of its cost-effectiveness and the potential for rapid expansion of local capacity.^5^ Additionally, the development and utilisation of local trainers ensure that the curriculum content is culturally relevant and applicable.^6^ Despite these benefits, there are challenges associated with the TOT approach. One is the concern over the ‘wastage’ or low utilisation rate of TOT graduates; some studies have reported that 30% − 50% of TOT trainees did not deliver any training after the TOT.^5,6^ There are also issues with ‘fidelity of implementation’ – the dilution and distortion of core messages and information as the programme is cascaded downstream because trainers fail to adhere to the curriculum and training protocols;^5,6^ and also perhaps because documentation of the curriculum does not provide sufficient details to ensure standardisation. Furthermore, qualifications of TOT participants may be inadequate if their selection does not take into account future availability to train and their defined roles in the programme, in addition to technical competency.

Great variations of TOT methodology have been described, ranging from didactic presentations to group discussions and role-play.^7^ Some TOT workshops put equal emphasis on curriculum content learning and facilitation skills development whilst others focus exclusively on the former. Although there is no consensus regarding the ideal TOT model,^4^ there has been an increasing trend from traditional passive teaching methods toward active methods that involve the students in both the teaching and learning process.^8^ In this paper, we describe a teachback-based TOT model for building in-country and regional capacity in the implementation of laboratory quality management systems and, specifically, the SLMTA programme. We discuss how the SLMTA TOT strategy ensures high utilisation of TOT graduates and maintains the fidelity of implementation whilst facilitating the rapid scale-up and sustainability of the programme.

## Research method and design

### Teachback methodology

The SLMTA TOT uses the teachback methodology developed by Dr Gordon Pask in 1975.^9^ Teachback has been recognised as an effective method for educating and assessing learning for patients in clinical practice^10^ and has been adapted to the teaching of training skills to trainers as well. This intensive practice-based training approach requires participants to play the roles of both trainer and participant: they must teach the curriculum at the same time as they are learning the curriculum content. The SLMTA TOT is organised into three phases: (1) TOT facilitators, also called master trainers, teach the curriculum whilst modeling the learner-centred, interactive facilitation skills; (2) participants learn the curriculum content and practise teaching it back to other participants; and (3) master trainers provide feedback on the teachback performance.^11^

Feedback is the cornerstone of teachback methodology and follows a distinct protocol. It is given immediately at the end of each teachback session after the participants have had a chance to assess their own performance. Praise is always offered first, followed by suggestions for improvement. Effective feedback is positive, encouraging and specific, building upon each participant’s strengths. Comments must be constructive, focusing on areas that can be improved and behaviours that can be changed. Master trainers use standard feedback forms to guide them on what to look for during the teachback. Participants receive their feedback both orally and on the written feedback forms.^11^

### The SLMTA trainer’s toolkit

The SLMTA programme provides a training curriculum that embodies the principle of ‘learning by doing’; lectures are limited to only 20% of the total training time whilst 80% is demonstration, discussion, role play, simulation and hands-on practice. The curriculum comprises 44 activities, which teach participants to use more than 100 tools and job aids in performing 66 management tasks and routines. It requires a facilitation style that engages the participants actively in the learning process. Each TOT participant receives a trainer’s toolkit containing all the information necessary to teach the 44 SLMTA activities. In addition to the handouts, tools, worksheets and job aids, the toolkit provides detailed preparation instructions and a step-by-step teaching protocol, grounded in adult learning principles, for each activity. During the TOT, master trainers and participants are both required to adhere to the protocols prescribed in each activity. The only deviation allowed is their personal examples and stories for illustrating key concepts and creativity in designing the visual aids. This comprehensive toolkit, coupled with the rigorous teachback process, serves to preserve the fidelity of the programme as the participants deliver the training back home.

### SLMTA training-of-trainers workshop structure

The SLMTA TOT teaches participants to deliver the SLMTA curriculum effectively and to implement the programme appropriately. The TOT workshop lasts two weeks. Because of its intensive performance-based nature, the participant-to-facilitator ratio must not exceed eight to one; a typical TOT workshop has at least three master trainers for a maximum of 24 participants.

To ensure return on investment and a high utilisation rate of the TOT graduates, countries are asked to screen their participants carefully using set criteria. Ideal candidates are those who have participated in the SLMTA process and successfully implemented improvement projects in their laboratories. Additional qualifications include: (1) availability to train; (2) defined role to implement the programme as designated by the country’s Ministry of Health; (3) evidence of motivation; (4) excellent training and communication skills; (5) technical laboratory experience in a clinical setting; and (6) proven ability to manage a laboratory successfully.

It is critical to balance participants’ need to learn the unconventional curriculum from the master trainers first hand, with sufficient time to practise teaching each other. The 44 SLMTA activities are thus presented differently, depending on their level of complexity. Master trainers typically teach the 25 most difficult activities so as to ensure fidelity, whilst describing (instead of teaching) the 10 least complicated activities to the participants. Sixteen activities are assigned to participants to teach back. Some of these activities are so important that they are taught initially by master trainers and then assigned to participants for teachback. Table 1 lists the 44 activities in the curriculum and how they are presented during the TOT.

**TABLE 1:** Workshop activities and how they are presented.

Workshop Activities	How is each activity is learned in the TOT?		
	Taught by master trainers	Described by master trainers	Taught by participants in Teachback
**Introduction**			
1. Envision your dream laboratory	-	X	-
2. ‘My lab’ key message puzzles	-	X	-
**Cross-Cutting**			
3. Process mapping	X	-	-
4. Managing performance – the balanced scorecard	X	-	X
5. PDCA cycle as the improvement method	X	-	X
6. Workstation set-up (recurring across 10 modules)	X	-	-
7. What would you do? (recurring across 10 modules)	X	X	-
8. Planning improvement projects – master class	X	-	-
9. Reporting improvement projects	-	X	-
10. Conducting an SLMTA follow-up visit	X	-	-
**Module 1: Productivity management**			
11. Process + structure = outcome	X	-	-
12. Mapping out the floor plan of your laboratory	X	-	-
13. Redesigning the floor plan of your laboratory	X	-	-
14. Improving a problem floor plan	X	-	-
15. Making a cup of tea	X	-	-
16. Whisper down the alley	X	-	-
17. What are the benefits of a standardised process?	X	-	-
18. How do you assign personnel to tasks?	X	-	X
19. Creating a management calendar	X	-	X
20. Competency assessment	-	X	-
21. Planning and conducting a staff meeting	-	X	-
22. Creating a personnel file	-	-	X
**Module 2: Work area management**			-
23. Laboratory safety demonstrations	X	-	-
24. Assessing safety incidents	-	-	X
25. Conducting a safety audit	-	-	X
26. What did we see on the site visits?	-	X	-
**Module 3: Inventory management**			
27. Creating a list of supplies for a test	X	-	-
28. What is wrong with this storeroom?	X	-	
29. Did you receive what you ordered?	-	-	X
**Module 4: Procurement management**			
30. Forecasting and calculating ordering amounts	X	-	X
**Module 5: Maintenance of equipment**			
31. Creating a maintenance and QC log	X	-	X
32. Making a service call	X	-	-
**Module 6: Quality assurance**			
33. Using standard operating procedures	-	-	X
34. Is QC that important?	X	-	-
35. Is there more to QC than just plotting the data?	X	-	-
**Module 7: Specimen collection and processing**			
36. Specimen collection: phlebotomy role-play	-	X	-
37. Specimen management	-	X	-
38. Packaging specimens for shipment to referral sites	-	-	X
39. Tracking referral specimens	-	-	X
**Module 8: Laboratory testing**			
40. Validation of test results	-	-	X
41. Is the test report ready to be released?	-	-	X
**Module 9: Test result reporting**			
42. Customer service	-	-	X
43. Meet the clinician	-	X	-
**Module 10: Document and record management**			
44. Why was the outdated version used?	X	-	-

TOT, training-of-trainer; PDCA, Plan, Do, Check, Act; SLMTA, Strengthening Laboratory Management Toward Accreditation; QC, quality control.

The 35 activities taught or described by master trainers occur in plenary sessions. The teachback sessions, on the other hand, occur initially in small breakout groups in order to foster a nurturing and less intimidating environment. Each breakout group typically contains eight people, who are then further divided into four teams of two. These teams become teachback partners and remain together throughout the workshop for their teachback assignments. Each breakout group is led by a master trainer who serves as a mentor and coach for the group.

The 16 teachback activities are assigned evenly to the four teams within each breakout group: each team is responsible for teaching back a total of four activities. The first three are done within their small breakout groups and the fourth is done in plenary sessions to give participants the experience of teaching in a large-group setting. To allow time for preparation, teachback does not begin until the end of the first week of the two-week TOT workshop.

Specific times are allocated for coaching, usually at the end of each day. Coaching sessions last 30–60 minutes and allow teams to discuss their upcoming teachback assignments with their master trainers. Additional coaching sessions are available by appointment with master trainers. Teams then prepare for their assignments, often late into the evening.

### Final participant assessment

In addition to the feedback given immediately after each teachback presentation, master trainers meet with individual participants privately at the end of the TOT and provide assessment orally on their overall performance. A written report is also submitted to the participants and their organisations following the TOT. Irrespective of the assessment, all participants who meet the 100% participation requirements are allowed to graduate from the course.

### Training-of-trainers programme scale-up

Beginning in 2009, SLMTA TOTs have been conducted at the African Centre for Integrated Laboratory Training (ACILT) in Johannesburg, South Africa, enrolling participants from multiple countries. As the SLMTA programme spreads deeper within countries, more local trainers and implementers are needed. The ACILT-based TOT, which admits only two to four participants from each country, is no longer sufficient. To meet the demand, 12 countries and/or regions to date (Botswana, Dominican Republic, Ethiopia, Ghana, Kenya, Mozambique, Nigeria, Rwanda, South Africa, Tanzania, Vietnam/Cambodia and Zimbabwe) have hosted their own TOT workshops. Prerequisites for an in-country TOT include completion of at least one round of the SLMTA process (three SLMTA workshops with supervisory visits and audits), as well as a sound country roll-out plan that details how the increased capacity will be used effectively.

### Development of the master trainer cadre

SLMTA’s TOT strategy hinges on the availability of master trainers. With the expansion of the TOT programmes, the need for master trainers, who facilitate the TOT workshops and mentor future trainers, has escalated. Starting in 2011, an effort was made to increase the number of master trainers with a focus on building in-country capacity.

Master trainers have extensive experience in training, coaching and feedback as well as in implementing laboratory quality systems. They play a critical role in ensuring programme fidelity in the rapid scale-up of SLMTA around the world. There is a stringent selection and grooming process to create these master trainers. To be eligible for consideration, a candidate must be a certified SLMTA trainer and have implemented at least one round of SLMTA roll-out in-country. This includes teaching the entire series of SLMTA workshops, mentoring laboratories to implement improvement projects and conducting follow-up visits after each workshop. The candidate must be available and must be released by their employer to conduct the two-week long TOT. In addition, they must be engaged in all preparation tasks leading to the workshop. Finally, this candidate must be nominated by a master trainer and approved by his or her country’s SLMTA programme coordinator or office before being invited to a TOT for apprenticeship.

Master trainer candidates shoulder equal amounts of responsibility in the TOT under the watchful eye of master trainers. They participate fully in the pre-TOT planning sessions and serve as lead facilitators for activities assigned to them. They coach the teams to prepare for teachback and provide feedback afterwards. Candidates must be prepared to re-teach activities whenever a team fails to deliver their teachback activity effectively. They also provide input to the final assessment of each participant’s performance (both oral and written). In turn, they receive coaching and feedback from the master trainers throughout the TOT. At the end of the workshop, master trainers provide feedback and recommendations. Because of the apprenticeship model used to develop new master trainers, each TOT workshop can accommodate up to three master trainer candidates, with each master trainer mentoring one candidate.

### Training-of-trainers programme evaluation

An online survey was conducted in August 2013 in order to assess the effectiveness of the TOT strategy on building country capacity for programme scale-up. It included both open- and closed-ended questions to collect data on TOT graduates’ utilisation rates and on how well the TOT prepared them for programme implementation using a four-point Likert scale (extremely well; well; not sure; and not at all). The survey was sent to all 316 SLMTA TOT graduates from 25 countries who attended one of the 14 regional or in-country TOT workshops held between November 2009 and March 2013. One hundred and seventeen more recent TOT graduates were not surveyed because they would not yet have had enough time to deliver any SLMTA training.

To verify the TOT graduates’ survey data, a second survey was sent to the programme leaders in all 10 countries that had held a local TOT before March 2013 and therefore had large trainer populations. These countries’ SLMTA programme leaders were asked to provide data on the number of trainers that are still involved in SLMTA activities.

## Results

**FIGURE 1:**
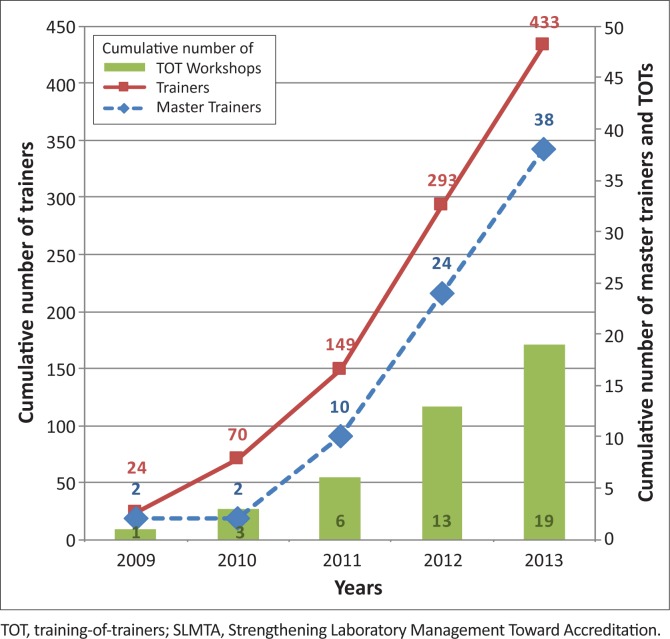
Cumulative number of SLMTA trainers, master trainers and TOTs 2009–2013.

Between November 2009 and November 2013, 8 regional and 11 local TOT workshops have been conducted, yielding a total of 433 trainers (Figure 1). To expand the programme in non-English speaking countries, Vietnam, Mozambique and the Dominican Republic each hosted a TOT dedicated to producing Vietnamese-, Portuguese- and Spanish-speaking trainers, respectively. To address the shortage of Francophone trainers, two French-language TOTs are being planned by Cameroon and Cote d’Ivoire.

**FIGURE 2:**
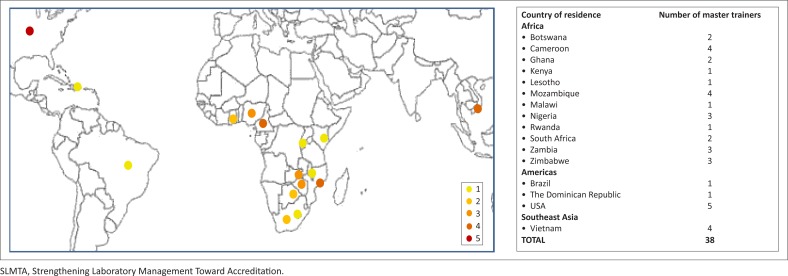
Global distribution of SLMTA master trainers.

Before 2011, there were only two master trainers, both based in the United States. As of the end of 2013, there were a total of 38 master trainers: 27 in Africa (71%), seven (18%) in the Americas (including two in Latin America) and four in Southeast Asia (11%) (Figures 1 and 2). The language portfolio of the master trainers now includes French (*n* = 5), Portuguese (*n* = 5), Vietnamese (*n* = 4) and Spanish (*n* = 2), in addition to English.

**FIGURE 3:**
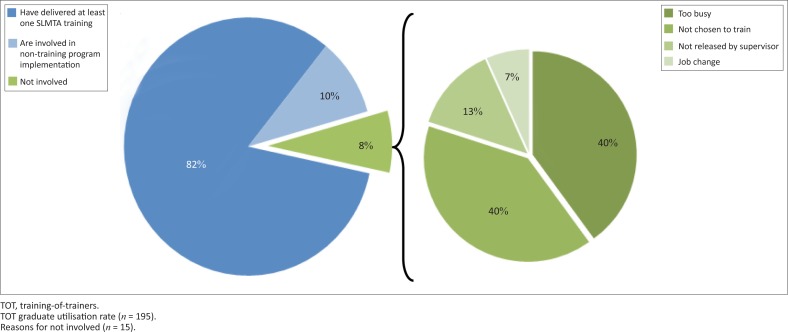
Results from the TOT Graduates Survey.

Of the 316 TOT graduates from November 2009 to March 2013, 195 (62%) returned the survey. Of the respondents, 160 (82%) have delivered at least one SLMTA training. An additional 19 (10%) are still involved in SLMTA programme activities such as mentoring and coordination, yielding a 92% utilisation rate. For the remaining 8% (*n* = 15) of the TOT graduates, reasons for non-involvement included being too busy, not being chosen to train, not being released by supervisors and changing jobs (Figure 3).

Of the 160 respondents who had facilitated SLMTA training(s), 59% (*n* = 94) stated that the TOT prepared them extremely well and 36% (*n* = 58) well. The remaining 5% (*n* = 8) of the respondents were not sure about the effectiveness of the TOT, viewed it as being not at all helpful, or did not respond. The 19 respondents who had been involved only in non-training aspects of programme implementation stated that the TOT had prepared them extremely well (*n* = 13; 69%) or well (*n* = 6; 31%).

All 10 country programme leaders returned the survey. Current involvement rates of their local TOT graduates (as of August 2013) ranged from 62% to 100%, with an overall 87% (*n* = 197) of TOT graduates still being involved in SLMTA activities (Table 2).

**TABLE 2:** Results from the country programme leader survey.

Country	Number of TOT graduates currently involved in SLMTA activities†	Total number of TOT graduates in country‡	Currently involved in SLMTA activities (%)
Cambodia	9	11	82
Ethiopia	28	31	90
Kenya	24	26	92
Mozambique	23	26	88
Nigeria	31	31	100
Rwanda	8	13	62
South Africa	21	23	91
Tanzania	21	24	88
Vietnam	14	17	82
Zimbabwe	18	24	75
**Total**	**197**	**226**	**87**

TOT, training-of-trainers; SLMTA, Strengthening Laboratory Management Toward Accreditation.

† As of August, 2013;

‡ As of March, 2013.

## Discussion

Since SLMTA TOT began in 2009, the SLMTA programme has expanded rapidly^1,2^ and many countries have rolled out the programme independently with little or no outside assistance. Evidence suggests that the SLMTA TOT strategy has been effective in building local capacity to accelerate programme expansion in multiple regions of the world,^2^ with 97% of the TOT graduates and 87% of the master trainers based in developing countries. Today, these cadres of master trainers, trainers and SLMTA-trained laboratory managers serve as the local champions of laboratory quality and activists in a global network that has kept the SLMTA ‘grassroots movement’ going.

High utilisation rates of the TOT graduates have been observed, at 92%. However, the relatively low response rate (62%) on the part of the TOT graduates may limit the generalisation of this observation. The low response rate can most likely be attributed to the short survey period, which lasted only two weeks, as well as the fact that an online survey was used which required access to the internet. In the second survey, however, all 10 countries’ programme leaders responded and they reported an average 87% utilisation rate, which is similar to the results from the first survey.

Fidelity of implementation is also critical as the programme expands and the cadre of trainers multiplies. The quality of training is more difficult to evaluate and was not measured directly in this study. However, evidence suggests that there has been no deterioration of implementation quality with programme expansion, as average audit improvement scores for laboratories implementing SLMTA in 2011–2013 were the same as those for laboratories implementing SLMTA during its initial year (2010), at 24%.^2^ Additionally, TOT participants reported overwhelmingly that the training was effective in preparing them to implement the programme. Several key elements have been built into the SLMTA TOT in an effort to ensure continued quality: (1) the SLMTA training toolkit comprehensively standardises training protocols and content, assisting trainers to teach the information consistently; (2) a highly structured and demanding TOT programme ensures that trainers are fully capable and confident in their training abilities; (3) a prescribed intensive implementation process fosters commitment, understanding and collaboration; and (4) a rigorous process is used for selecting and grooming master trainers, who are the gatekeepers for the quality of future trainers.

A collaborative south-to-south network has emerged. Eighty-seven per cent of the 38 master trainers reside in 15 developing countries across Africa, Southeast Asia and Latin America. These highly-skilled master trainers are often invited to conduct TOT workshops in countries other than their own. Notably, the depth of the African master trainer pool allowed the export of two master trainers to assist Latin America when it held its first regional SLMTA TOT in the summer of 2013.

Furthermore, most in-country TOTs provide a few slots for participants from other countries. This fosters networking and experience sharing, as well as assisting countries who are not ready to conduct their own TOT but do not want to wait for the next available TOT at ACILT. There are many examples where countries with SLMTA experience are aiding those without sufficient training capacity: Rwandan trainers travelled to Burundi to deliver workshops; Namibian participants went to Zimbabwe to be trained; the Dominican Republic enrolled participants from six Central American countries alongside its own people; and the African Field Epidemiology Network, headquartered in Uganda, delivered SLMTA training in the Caribbean Region. The globally-standardised SLMTA curriculum and common implementation process facilitate cross-border and cross-region technical assistance.

### Next steps

Casual observers of the programme have noted that the current geographic distribution of the master trainers does not reflect the size and maturity of country programmes (Table 3). Of the seven countries with the largest number of laboratories enrolled in SLMTA,^2^ three (Ethiopia, Uganda and Tanzania) have no master trainers, whilst Kenya, with the most rounds of implementation (six), has only one master trainer. Lacking master trainers does not necessarily correspond to the quality of a country’s programme, since SLMTA is implemented by trainers rather than master trainers; nevertheless, going forward it seems prudent that these countries should be encouraged to build a pool of local master trainers. This will not only reduce costs for future TOTs by eliminating the need to bring in international master trainers, but will also motivate in-country trainers to be involved in more programme activities so they may be eligible to become master trainers.

**TABLE 3:** Number of master trainers in countries with the most enrolled SLMTA laboratories.

Country	Number of enrolled laboratories	Number of SLMTA rounds implemented	Number of master trainers
Ethiopia	81	5	0
Uganda	76	2	0
Kenya	57	6	1
Malawi	41	2	1
Zimbabwe	32	3	3
Nigeria	30	2	3
Tanzania	30	2	0

SLMTA, Strengthening Laboratory Management Toward Accreditation.

## Conclusion

SLMTA has helped transform the laboratory landscape in resource-limited countries worldwide.^1^ Through the careful execution of a deliberate TOT strategy, the programme has focused on building local capacity to accelerate the breadth and depth of programme spread. Rapid programme scale-up has been achieved with continued high quality results,^2^ using a strategy of centralised and in-country TOTs, in-country workshops and implementation at the laboratory level. Strict maintenance of training and qualification standards has been key to the success of the strategy. The growing pool of motivated and skilled local implementers will be critical with regard to sustaining the on-going quality improvement and accreditation drives in resource-limited settings.

Skills acquired through the SLMTA TOT, such as programme planning, mentoring and training facilitation, will reach beyond SLMTA to make participants become more effective managers and mentors. Moreover, it has been suggested that SLMTA be ‘adapted for clinical settings in developing countries, with a goal towards overall hospital accreditation’.^1^ Based on the success of the SLMTA TOT model, we would recommend that other programmes consider adopting the model to build local capacity for rapid programme expansion whilst maintaining programme quality.
